# “Low-age, low-frequency” lung cancer screening strategies maybe adaptable to the situation in China

**DOI:** 10.1186/s12893-023-02279-x

**Published:** 2023-12-08

**Authors:** Peixuan Hu, Xiaozhen Song, Xiaowu Fan, Yunpeng Zhu, Xiangning Fu, Shengling Fu

**Affiliations:** 1 Department of Thoracic Surgery, Tongji Hospital, Tongji Medical College, HuaZhong University of Science and Technology, Wuhan, Hubei 430030 People’s Republic of China; 2 The Second Clinical School, Tongji Medical College, HuaZhong University of Science and Technology, Wuhan, Hubei 430030 People’s Republic of China

**Keywords:** Lung cancer, Screening, Ground Glass Opacity

## Abstract

**Background:**

The object was to compare changes in patients undergoing lung surgery before and after COVID-19 outbreak, and to explore the impact of COVID-19 on lung surgery and its coping strategies.

**Method:**

A retrospective review of patients undergoing thoracic surgery at a single institution was conducted. Group A included patients treated between January 23, 2019, and January 23, 2020, while Group B included patients treated between June 1, 2020, and June 1, 2021, at our center. We compared the reasons of seeking medical treatment, the general characteristics of patients, imaging features, pathological features, surgical methods and postoperative recovery.

**Result:**

Compared to Group A, the number of patients with pulmonary nodules screened by routine check-up increased in Group B (57.6% vs 46.9%, *p* < 0.05). Female patient increased (55.2%vs 44.7%). Patient without smoking history or with family history of lung cancer increased (70.7% vs 60.7%) (10.1%vs 7.8%). Early stage lung cancer increased. Lobectomy decreased (53.4% vs 64.1%). Segmental resection increased (33.3% vs 12.7%). Patients without postoperative comorbidities increased (96.1%vs 85.7%).

In the case of patients with Ground Glass Opacity(GGO), their age was comparatively lower (52 ± 9.9 vs. 55 ± 10.7), the female patients increased, patient without smoking history, tumor history, family history of tumor increased, small GGO increased. Lobectomy decreased (35.2% vs 49.7%). Segmental resection increased (49.6% vs 21.2%). Patients without postoperative comorbidities increased (96.5% vs 87.4%).

**Conclusion:**

Since COVID-19 outbreak, more young, non-smoking, female lung cancers, more Ground Glass Opacity, none high risk patients have been detected through screening, suggesting that our current screening criteria for lung cancer may need to be revised. Higher requirements, including the selection of the timing of nodular surgery, surgical methods were put forward for thoracic surgeons’ skills.

**Supplementary Information:**

The online version contains supplementary material available at 10.1186/s12893-023-02279-x.

## Introduction

Lung cancer is the most frequently diagnosed cancer all over the world, with about 1.8 million new lung cancer cases occur every year [1]. Lung cancer accounts for 12.8% of the total cancer cases and 17.8% of the total cancer deaths. Subjects who had symptoms were more likely to have been diagnosed with stage III (34.4%) or IV (28.7%) disease than those who did not have symptoms [2]. Most lung cancer cases weren’t diagnosed until advanced stage, which leads to poor prognosis. The 5-year survival rate of non-small cell lung cancer was only 26% [3].Studies have shown that the 5-year survival rate of stage IA, IB and IIA lung cancer is 83%, 71% and 57%, respectively [4]. In the absence of lung cancer screening, early-stage lung cancer may only be incidentally detected through imaging examinations performed for other purposes.

Low-dose computed tomography(LDCT) was once considered to be more effective than laboratory examination in the diagnosis of COVID-19 [5], so the COVID-19 pandemic in Wuhan from January 2020 to June 2021 popularized LDCT which diagnosed many early lung cancer. We compared the changes of patient composition of pulmonary surgery 1 year before and after the COVID-19 outbreak, and compared the surgical methods and postoperative recovery of various patients. We also compared the ratio of ground-glass opacities (GGO) to solid nodules among lung cancer patients presenting for treatment before and after the COVID-19 outbreak. The study was conducted at Tongji Hospital in Wuhan, one of the largest thoracic surgery center in China to explore the impact of the outbreak of COVID-19 in Wuhan on the composition of patients with surgically treated pulmonary nodules and surgical management.

## Method

### Setting and study design

Patients (*n* = 4645) diagnosed with lung cancer or benign lung tumors were included in this study. The patients were evaluated at Tongji Hospital, Huazhong University of Science & Technology, during two time periods: from January 23, 2019, to January 23, 2020 (Group A, *n* = 2033), and from June 1, 2020, to June 1, 2021 (Group B, *n* = 2612). In Group A, there were 1234 cases of solid nodules, 754 cases of ground-glass opacities (GGO), and 45 cases of cavities. In Group B, there were 1277 cases of solid nodules, 1314 cases of GGO, and 21 cases of cavities. The study was conducted in Wuhan, where COVID-19 first breakout in China. 2020-01-23 is the time for Wuhan to be closed and 2020-06-01 is the time to be unsealed. Group A consisted of patients from the entire year before the lockdown in Wuhan, while Group B comprised patients from the entire year after the lifting of the lockdown in Wuhan. Only patients who underwent surgical treatment of pulmonary lesions were selected for this study. Medical record data of whom meet the above inclusion criteria were collected, and retrospective analysis could be done based on these data.

### Data collection

The following information of these patients were collected: gender, age, smoking history, family history of tumor, previous tumor history, combined diseases. Radiographic features and pathological staging contain the following information: number of nodules found by CT in each patient, radiological tumor size, radiological size of solid component, consolidation/tumor ratio (CTR), pathologic diagnosis, TNM stage. We also collected the symptoms and signs of patients, the surgical method, postoperative discharge time, perioperative complications and postoperative complications.

### Statistical analysis

Continuous variables subject to normal distribution were expressed as mean and standard deviation, continuous variables not subject to normal distribution were expressed as median and quartile, and classified variables were expressed as constituent ratio, total number, percentage. Comparison of categoric variables were performed with the chi-square test, continuous variables subject to normal distribution were analyzed with the Student’s t-test as appropriate. Rank sum test is for continuous variables that do not obey normal distribution. A two-tailed *P*-value 0.05 was considered statistically significant. Data processing and statistical analyses were performed using the statistical package for the social sciences (SPSS) 26.0 statistical program.

The study was approved by the Medical Ethics Committee of Tongji Hospital, Tongji Medical College, Huazhong University of Science and Technology (December 23, 2022). Before each patient’s surgery, we have obtained their consent to use their clinical data for scientific research.

## Result

### Pre visit symptoms

In group A and B, 2033and 2612 patients with lung cancer or benign tumor received surgical treatment. The symptoms and main complaint of patients before treatment were analyzed in order to find out whether the proportion of asymptomatic early lung cancer was changed. As shown in Table 1, routine check-up, cough or expectoration or hemoptysis, chest tightness or chest pain, dyspnea, hoarseness, other symptoms and accidental discovery accounted for 46.9%, 19.9%, 13.6%, 1.3%, 0.5%, 9.4% and 8.4% in group A, respectively. The corresponding proportion in group B were 57.6%, 17.3%, 9.2%, 0.8%, 0, 6.5% and 8.6% respectively (*P* < 0.05). 46.9% of patients in group A and 57.6% in group B found lung cancer through routine check-up (*P* < 0.05).

**Table 1:** Pre-visit symptoms

Variables	Group A *n* = 2033 N(%)	Group B *n* = 2612 N(%)	*P* value
CLINICAL SYMPTOMS AND SIGNS BEFORE DIAGNOSIS			*P* < 0.05
Routine check-up	954 (46.9)	1505 (57.6)	
Cough or expectoration or hemoptysis	404 (19.9)	452 (17.3)	
Chest tightness or chest pain	276 (13.6)	240 (9.2)	
Dyspnea	26 (1.3)	21 (0.8)	
Hoarseness	10 (0.5)	0 (0)	
Other symptoms	191 (9.4)	170 (6.5)	
Found occasionally	172 (8.4)	224 (8.6)	

### Patient characteristics of pulmonary lesions

The proportion of male in group A and group B were 55.3% and 44.8% (Table 2, *P* < 0.05), verified by nonparametric test. The proportion of patient who never smoked, once smoked but has quit for more than 15 days, never quit smoking were 60.7%, 25.9% and 13.4% in group A and 70.7%,21.0% and 8.3% in group B, respectively (*P* < 0.05). There were more lung cancer patients who had never smoked in group B than those in group A. The proportion of patient who had no family history of tumor, had family history of lung cancer, had family history of other tumor are 80.4%,7.8%,11.8% in group A and 79.6%,10.1%,10.3% in group B, respectively (*P* < 0.05). More patients in group B had a family history of lung cancer than in group A. The proportion of patient with no previous history of tumor, previous history of lung cancer, previous history of other tumors were 87.7%,1.7%, 10.6% in group A, and 92.1%, 1.0% and 6.9% in group B, respectively (*P* < 0.05). Group B had more patients with no history of cancer than group A.

**Table 2:** Patient characteristics of pulmonary lesions

Variables	Group A *n* = 2033 N(%)or median	Group B *n* = 2612 N(%)or median	*P* value
AGE			*P* = 0.105
Median	57	56	
GENDER			*P* < 0.05
Male	1124 (55.3)	1170 (44.8)	
Female	909 (44.7)	1442 (55.2)	
SMOKING HISTORY			*P* < 0.05
Never	1234 (60.7)	1846 (70.7)	
Quit smoking	526 (25.9)	549 (21.0)	
Not quitting smoking	273 (13.4)	217 (8.3)	
FAMILY HISTORY OF TUMOR			*P* < 0.05
None	1634 (80.4)	2079(79.6)	
Lung cancer	159 (7.8)	264 (10.1)	
Other cancer	240 (11.8)	269 (10.3)	
PREVIOUS HISTORY OF TUMOR			*P* < 0.05
None	1783 (87.7)	2405 (92.1)	
Lung cancer	35 (1.7)	26 (1.0)	
Other cancer	215 (10.6)	181 (6.9)	
COMORBIDITIES			*P* < 0.05
None	1307(64.3)	1794(68.7)	
Hypertension	468 (23.0)	497 (19.0)	
Diabetes	122 (6.0)	159 (6.1)	
Coronary heart disease	36 (1.8)	70 (2.7)	
Chronic pulmonary disease	100 (5.0)	92 (3.5)	
HIGH RISK GROUP OF LUNG CANCER	823 (40.5)	744 (28.5)	*P* < 0.05

High-risk groups according to NCCN: 1-The first group consists of individuals aged 55 to 74 years with a smoking history of 30 or more pack-years who currently smoke or, if former smokers, have quit within the last 15 years. 2-The second category includes individuals aged 50 years or older with a smoking history of 20 or more pack-years and at least one additional risk factor. These additional risk factors may include a personal history of cancer or lung disease, a family history of lung cancer, exposure to radon, and occupational exposure to carcinogens

In Group A, patients’ conditions were as follows: no other disease in 64.3%, while 23.0% had hypertension, 6.0% had diabetes, 1.8% had coronary heart disease, and 5.0% had chronic pulmonary disease. These proportions were 68.7%, 19.0%,6.1%, 2.7% and 3.5% in group B (*P* < 0.05), respectively. Compared with group A, the proportion of patients without underlying diseases and patients with chronic pulmonary disease was significantly lower in group B.

According to the National Comprehensive Cancer Network (NCCN) clinical practice guidline, the high risk groups are [6]: The first group were individuals aged 55 to 74 years with a 30 or more pack-year history of smoking tobacco who currently smoke or, if former smoker, have quit within 15 years. The second category is those aged 50 years or older with a 20 or more pack-year history of smoking tobacco and with one additional risk factor. These additional risk factors include personal history of cancer or lung disease, family history of lung cancer, radon exposure, and occupational exposure to carcinogens. The high-risk group accounted less in group B than that in group A (40.5% vs 28.5%, *P* < 0.05).

### Imaging features and pathological characteristics of pulmonary lesions

In terms of imaging classification, the proportion of pure GGO, part solid nodules, pure solid nodules, and cavity were 24.5%, 12.6%, 60.7%, 2.2% in group A, and 28.7%, 21.6%, 48.9%, and 0.8% in group B, respectively (Table 3, *P* < 0.05). GGOs’ constituent ratio is larger in group B than in group A.

**Table 3:** Imaging features and pathological characteristics of pulmonary lesions

Variables	Group A *n* = 2033 N(%)	Group B *n* = 2612 N(%)	*P* value
RADIOLOGICAL TUMOR TYPE			*P* < 0.05
Pure GGO	498 (24.5)	750 (28.7)	
Part GGO	256 (12.6)	564 (21.6)	
Pure solid	1234 (60.7)	1277 (48.9)	
cavity	45 (2.2)	21 (0.8)	
PATHOLOGICAL CLASSIFICATION			*P* < 0.05
AAH	30 (1.5)	44 (1.7)	
AIS	169 (8.3)	232 (8.9)	
MIA	155 (7.6)	465 (17.8)	
IAC	854 (42)	1356 (51.9)	
Squamous cell carcinoma	228 (11.2)	196 (7.5)	
Small cell lung cancer	22 (1.1)	8 (0.3)	
Metastatic carcinoma	18 (0.9)	5 (0.2)	
Neuroendocrine tumor	14 (0.7)	3 (0.1)	
Adenosquamous carcinoma	4 (0.2)	5 (0.2)	
Big cell lung cancer	4 (0.2)	3 (0.1)	
Other malignant tumors	67 (3.3)	21 (0.8)	
Benign	468 (23)	274 (10.5)	
TNM STAGING			*P* < 0.05
0	246 (12.1)	253(9.7)	
IA1	391 (19.2)	875 (33.5)	
IA2	449 (22.1)	611 (23.4)	
IA3	205 (10.1)	193 (7.4)	
IB	234 (11.5)	241 (9.2)	
IIA	47 (2.3)	34 (1.3)	
IIB	122 (6)	128 (4.9)	
IIIA	252 (12.4)	201 (7.7)	
IIIB	79 (3.9)	26 (1)	
IV	8 (0.4)	50 (1.9)	

*GGO* Ground glass opacity, *AAH* Atypical adenomatous hyperplasia, *AIS* Adenocarcinoma in situ, *MIA* Microinvasive adenocarcinoma, *IAC* Invasive adenocarcinoma

The constituent ratio for Atypical Adenomatous Hyperplasia (AAH), Adenocariconoma in situ (AIS), Microinvasive Adenocariconoma (MIA) were 1.5%, 8.3%, 7.6% in group A, and 1.7%、8.9%、17.8% in group B (*P* < 0.05), respectively, MIA was higher (17.8% vs 7.6%, *P* < 0.05) and squamous cell carcinoma was lower (7.5% vs 11.3%, *P* < 0.05) in group B than those in group A. In addition, The proportion of benign lung lesions, large cell carcinoma, small cell carcinoma, squamous cell carcinoma, lung metastasis and other types of lung cancer in group B was less than those in group A (*P* < 0.05).

Consistent with imaging features and pathological types, the proportions of stage 0, IA1, IA2, IA3, IB were 12.1%, 19.2%, 22.1%, 10.1%, 11.5% in group A, and 9.7%, 33.5%, 23.4%, 7.4%,9.2% in group B, respectively (*P* < 0.05). The proportion of stage 0 and stage I lung cancer in group B was higher than that in group A (83.2% vs 75%, *P* < 0.05) .

### Characteristics of ground-glass opacity patients

GGO is a nonspecific radiologic finding defined as hazy opacity that does not obscure underlying bronchial structures or pulmonary vessels at HRCT [7]. Considering the significant increase in the number of GGO detected after the COVID-19 pandemic, we further analyzed the patients with GGO on imaging.

As shown in Table 4, the median ages of group A and B were 55 and 52 years, respectively (*P* < 0.05), and the proportion of female in group B was significantly higher than that in group A (48.8% vs 68.0%, *P* < 0.05). In terms of smoking history, the proportion of patients who never smoked, quit smoking and did not quit smoking was 79.1%, 14.6%, 6.3% in group A and 84.3%, 11.2%, 4.5% in group B, respectively (*P* < 0.05), the proportion of GGO patients who never smoked increased. (79.1% vs 84.3%, *P* < 0.05). In terms of previous tumor history, the proportions of no tumor history, lung cancer tumor history and other tumor history were 80.6%, 1.5%, 17.9% in group A, and 87.2%, 1.2%, 11.6% in group B, respectively. The proportion of patients without previous tumor history was higher in group B than that in group A (87.2% vs 80.6%, *P* < 0.05).

**Table 4:** Characteristics of GGO patients

Variables	GGO in Group A *n* = 601 N(%)or Median	GGO in Group B *n* = 1320 N(%)or Median	*P* value
AGE			*P* < 0.05
Median	55	52	
GENDER			*P* < 0.05
Male	308 (51.2)	422 (32.0)	
Female	293 (48.8)	898 (68.0)	
SMOKING HISTORY			*P* < 0.05
Never	475 (79.1)	1113 (84.3)	
Quit smoking	88 (14.6)	148 (11.2)	
Not quitting smoking	38 (6.3)	59 (4.5)	
FAMILY HISTORY OF TUMOR			*P* = 0.134
None	444 (73.9)	1002 (75.9)	
Lung cancer	72 (12.0)	173 (13.1)	
Other cancer	85 (14.1)	145 (11.0)	
PREVIOUS HISTORY OF TUMOR			*P* < 0.05
None	484 (80.6)	1151 (87.2)	
Lung cancer	9 (1.5)	16 (1.2)	
Other cancer	108 (17.9)	153 (11.6)	
COMORBIDITIES			*P* < 0.05
None	410 (68.2)	1011 (76.6)	
Hypertension	103 (17.1)	130 (9.8)	
Diabetes	28 (4.6)	87 (6.6)	
Coronary heart disease	11 (1.9)	22 (1.7)	
Chronic pulmonary disease	49 (8.2)	70 (5.3)	

For patients with underlying diseases, the proportion of no comorbidities, hypertension, diabetes, coronary heart disease, chronic pulmonary disease was 68.2%, 17.1%, 4.6%, 1.9% and 8.2% in group A, and 76.6%, 9.8%, 6.6%, 1.7%, 5.3% in group B, respectively. The proportion of patients without underlying diseases in group B was higher than that in group A. (76.6% vs 68.2%, *P* < 0.05).

### Imaging features and pathological characteristics of of patients with ground-glass opacity

GGO with diameter greater than 5 mm were included. As shown in Table 5, compared with group A, the proportion of single nodule in group B increased (88.3% vs 76.9%, *P* < 0.05), while the proportion of multiple nodules decreased. The tumor size of GGO did not obey the normal distribution, so the nonparametric test of independent samples was used, the median tumor size of GGO was 11 mm in group A and 12 mm in group B (*P* < 0.05) and the median size of solid components was 9 mm in group A and 7 mm in group B (*P* < 0.05). CTR, which is defined as the ratio of solid portion size to total size of GGO, was considered to be a simple and useful tool to predict tumor invasiveness and prognosis in patients with with GGO [8]. In GGO patients of group A, the constituent ratios of CTR = 0, 0 < CTR < 0.5, 0.5 ≤ CTR < 1 were 77.0%, 10.5% and 12.5%, respectively. These proportions were 83.7%, 8.3% and 8% in group B, respectively (*P* < 0.05), which reflected the trend of pGGO patients increased significantly (41.4% vs 28.1%) after COVID-19 pandemic.

**Table 5:** Imaging features and pathological characteristics of patients with GGOs

Variables	GGO in Group A *n* = 601 N(%)or Median	GGO in Group B *n* = 1320 N(%)or Median	*P* value
NO. OF LESIONS, DETECTED PER PATIENT			*P* < 0.05
1	462 (76.9)	1165 (88.3)	
2	108 (18)	113 (8.6)	
3	25 (4.2)	30 (2.3)	
≥ 4	6 (1.0)	12 (0.9)	
RADIOLOGICAL SIZE OF SOLID COMPONENT (MM)			*P* < 0.05
Median	9	7	
RADIOLOGICAL TUMOR SIZE (MM)			*P* < 0.05
Median	11	12	
CTR			*P* < 0.05
0	463 (77.0)	1105 (83.7)	
> 0, < 0.5	63 (10.5)	110 (8.3)	
≥ 0.5, < 1	75 (12.5)	105 (8)	
PATHOLOGICAL STAGE			*P* < 0.05
AAH	16 (2.6)	58 (4.4)	
AIS	139 (23.2)	210 (15.9)	
MIA	121 (20.1)	432 (32.7)	
IAC	240 (40.1)	540 (41.0)	
Squamous cell carcinoma	3 (0.5)	3 (0.2)	
Small cell lung cancer	0 (0)	3 (0.2)	
Metastatic cancer	2 (0.3)	0 (0)	
Other malignant tumors	4 (0.6)	1 (0.1)	
Benign	76 (12.6)	73 (5.5)	
TNM CLASSIFICATION			*P* < 0.05
0	150 (25)	246 (18.6)	
IA1	217 (36.1)	611 (46.3)	
IA2	146 (24.3)	332 (25.1)	
IA3	36 (6)	71 (5.4)	
IB	37 (6.2)	46 (3.5)	
IIA	0 (0)	1 (0.1)	
IIB	5 (0.8)	9 (0.7)	
IIIA	10 (1.6)	4 (0.3)	

*NO.* number, *AAH* atypical adenomatous hyperplasia, *AIS* adenocarcinoma in situ, *MIA* microinvasive adenocarcinoma, *IAC* invasive adenocarcinoma

The constituent ratios of AAH, AIS, MIA, invasive adenocariconoma (IAC), squamous cell carcinoma, small cell lung cancer, metastatic cancer, other malignancies and benign lesions were 2.6%, 23.2%, 20.1%, 40.1v, 0.5%, 0, 0.3%, 0.6%, 12.6% in group A, and 4.4%, 15.9%, 32.7%, 41.0%, 0.2%,0.2%, 0, 0.1%, 5.5% in group B, respectively (*P* < 0.05), which showed a significant increase in MIA and a decrease in squamous cell carcinoma and benign lesions after the COVID-19 outbreak.

The constituent ratios of stage 0, IA1, IA2, IA3, IB, IIA, IIB, IIIA of GGO patients were 25.0%, 36.1%, 24.3%, 6.0%, 6.2%, 0, 0.8%, 1.6% in group A, and 18.6%, 46.3%, 25.1%, 5.4%, 3.5%, 0.1%, 0.7%, 0.3% in group B, respectively (*P* < 0.05). After the outbreak of COVID-19, the proportion of GGO patients with stage 0 decreased, the proportion of IA1 increased significantly, and the proportion of IB to IIIA stages decreased, suggesting that more and more patients with early lung cancer presenting with GGO were found after the outbreak of COVID-19.

### Surgical method

Among all patients with pulmonary lesions, as shown in Table 6, the proportion of patients who underwent pneumonectomy, sleeve resection, lobectomy, segmental resection, wedge resection, electromagnetic navigation bronchoscope guided microwave ablation (ENB-MWA), electromagnetic navigation bronchoscopy (ENB) combined with thoracoscopic surgery (VATS) was 0.5%, 3.1%, 64.1%, 12.7%, 16.6%, 1.8%, 1.2% in group A and 0.5%, 2.1%, 53.4%, 33.3%, 8.2%, 1.5%, 1.0% in group B, respectively (*P* < 0.05). Among patients with GGO, the constituent ratios of sleeve resection, lobectomy, segmentectomy, wedge resection, ENB-MWA, ENB combined with VATS were 0.6%, 49.7%, 21.1%, 18.5%, 6.2%, 3.9% in group A and 1.4%, 35.2%, 49.6%, 8.9%, 3.0%, 1.9% in group B, respectively (*P* < 0.05). Consistent with the pathological characteristics and staging of pulmonary nodules, wedge resection and lobectomy decreased significantly after the outbreak of COVID - 19, while segmental resection increased significantly, accounting for almost half of GGO patients’ surgical procedures. In the meantime, the proportion of multiple nodules decreased, so did the proportion of pulmonary nodules ablation by ENB-MWA or ENB combined with VATS decreased.

**Table 6:** Surgical method

Variables	Group A *n* = 2033 N(%)	Group B *n* = 2612 N(%)	*P* value
SURGICAL METHODS FOR LUNG CANCER AND BENIGN TUMOUR			*P* < 0.05
pneumonectomy	11 (0.5)	13 (0.5)	
sleeve resection	63 (3.1)	55 (2.1)	
lobectomy	1303 (64.1)	1395 (53.4)	
segmentectomy	258 (12.7)	870 (33.3)	
wedge resection	337 (16.6)	214 (8.2)	
ENB-MWA	37 (1.8)	39 (1.5)	
ENB combined with VATS	24 (1.2)	26 (1.0)	
SURGICAL METHODS OF GGO	GGO in Group A *n* = 601 N(%)	GGO in Group B *n* = 1320 N(%)	*P* < 0.05
sleeve resection	4 (0.6)	18 (1.4)	
lobectomy	299 (49.7)	465 (35.2)	
segmentectomy	127 (21.1)	655 (49.6)	
wedge resection	111 (18.5)	117 (8.9)	
ENB-MWA	37 (6.2)	40 (3.0)	
ENB combined with VATS	23 (3.9)	25 (1.9)	

*ENB* electromagnetic navigation bronchoscope, *ENB-MWA* electromagnetic navigation bronchoscope-guided microwave ablation, *VATS* video assisted thoracic surgery

### Postoperative recovery

As shown in Table 7, for patients with pulmonary lesions, the proportion of patients in group A and group B without postoperative complications was 85.7% and 96.2% (*P* < 0.05), respectively, suggesting that the safety of surgery has been improved after the outbreak of COVID-19. The results were similar for patients with pulmonary GGO.

**Table 7:** Postoperative recovery

Variables	Group A *n* = 2033 N(%)	Group B *n* = 2612 N(%)	*P* value
COMPLICATIONS			*P* < 0.05
None	1742 (85.7)	2512 (96.2)	
Pleural effusion	122 (6)	50 (1.9)	
Pneumothorax	83 (4.1)	31 (1.2)	
Pulmonary infection	21 (1)	0 (0)	
Pleural effusion combined with pneumothorax	37 (1.8)	13 (0.5)	
Pulmonary infection combined with other complications	26 (1.3)	3 (0.1)	
Chylothorax	2 (0.1)	3 (0.1)	
COMPLICATIONS OF GGO	GGO in Group A *n* = 601 N(%)	GGO in Group B *n* = 1320 N(%)	*P* < 0.05
None	524 (87.3)	1272 (96.4)	
Pleural effusion	34 (5.6)	24 (1.8)	
Pneumothorax	23 (3.8)	15 (1.1)	
Pulmonary infection	10 (1.6)	1 (0.1)	
Pleural effusion combined with pneumothorax	3 (0.5)	4 (0.3)	
Pulmonary infection combined with other complications	6 (1)	3 (0.2)	
Chylothorax	1 (0.2)	1 (0.1)	

### Comment

In this study, we compared the general information, radiographic features, pathological types, tumor stages, surgical methods, and postoperative recovery of patients with pulmonary lesions at one of China’s largest thoracic surgery centers 1 year before and 1 year after the COVID-19 pandemic. A similar assessment of patients with Ground-glass Opacity (GGO) was conducted due to the significant increase in the proportion of patients with GGO following the outbreak of COVID-19.

Early detection and therapeutic intervention of early lung cancers is an important opportunity to reduce the total lung cancer mortality. So the NCCN recommends screening for lung cancer in people 55 to 74 years of age with 30 pack-years of smoking history [6]. The NCCN considers age less than 50 years and smoking history lower than 20 pack-year as low risk, which is not recommended for lung cancer screening [6]. And most guidelines recommend annual LDCT screening for lung cancer in high-risk populations. However, following the COVID-19 outbreak, we found more non-smoking, younger, early-stage lung cancer patients who did not have risk factors for lung cancer, with a major increase in MIA. So young, non-smoking women, traditionally considered “low risk for lung cancer,” are actually at greater risk of developing lung cancer.

COVID-19 pandemic prompted LDCT examination. At the beginning of the COVID-19 outbreak, LDCT has been reported to be more sensitive for COVID-19 diagnosis than laboratory tests [5]. With the widespread use of LDCT, an increasing number of asymptomatic early lung cancers have been detected and diagnosed [9–11]. In this study, there was an increase (46.9% vs 57.6%) in the number of lung cancers detected by routine check-up. After the outbreak of COVID-19, the median age was 56 for lung cancer patients and 52 for GGO patients. The proportion of early-stage lung cancers presenting with GGO increased significantly after the COVID-19 outbreak (37.1% vs. 50.2%). The GGO component indicated that the malignant degree of the tumor was not high, the tumor grew slowly, and the prognosis was generally good.

The early lung cancer presenting with GGO is mostly female, and the average age at diagnosis is significantly lower than the current screening criteria. Most patients have no risk factors for lung cancer, which is proved by our data. According to the current screening criteria for lung cancer, most patients with GGO lung cancer do not meet the screening criteria for lung cancer. Our study also shows that these individuals with low risk factors should also be paid attention to, and more individuals with low risk of lung cancer need to be included in lung cancer screening. However, based on the need for cost-effectiveness, it is worth discussing which patients should be included, at what age and how often they should be screened for lung cancer.

Therefore, we propose the following risk factor-based “low-age, low-frequency” lung cancer screening strategies. Earlier initial screening age is helpful to achieve the goal of early detection of early lung cancer, and the longer interval LDCT follow-up strategy can reduce the CT radiation dose and cost. Since GGO-type lung cancer is common in LDCT screening, the possibility of tumor progression caused by prolonged follow-up interval is small, and the new GGO that appears in a longer follow-up interval is also curable to a large extent, without affecting the survival rate of patients. We recommend that the first screening for lung cancer be as early as age 30. For people less than 50 years of age, if the initial CT is negative, the screening interval can be set to 5–10 years. For people aged 50 to 60, the interval between screening can be 3 to 5 years depending on whether there are high risk factors. For people aged 60–70, the interval between screening can be 2–3 years depending on the presence of risk factors. For people aged ≥70, the screening interval is 2 years.

By analyzing the pathological types and stages, we found the proportion of MIA significantly increased, the proportion of stage IA1 adenocarcinoma significantly increased and stage IB, IIA, IIB, IIIA and IIIB lung cancers all showed a decreasing trend after COVID-19 outbreak. Sub lobectomy is generally used for early lung cancer, segmentectomy leads to comparable overall survival (OS) and recurrence-free survival (RFS) as a lobectomy [12]. Corresponding to the stage of lung cancer, the proportion of lobectomy decreased (64.1% VS 53.45%) and segmentectomy increased (12.7% vs 33.3%) for pulmonary lesions after COVID-19 outbreak. An analysis of lung GGO procedures yielded similar results, except that segmentectomy accounted for almost half of the procedures. More early stage lung cancer posed great challenges to thoracic surgeons, such as the choice of surgical timing, the treatment of multiple nodules, and excision technique which technically put forward higher requirements for thoracic surgeons. We have used ENB to locate and ablate multiple nodules since October 2018 [13]. We found an increase in solitary pulmonary nodules and a decrease in multiple pulmonary nodules following the outbreak of COVID-19, resulting in a decrease in the ablation or localization of pulmonary nodules under ENB.

Changes in the proportion of surgical ways may lead to changes in surgical safety. In the comparison of postoperative recovery of pulmonary lesions, patients without complications increased (85.7% VS 96.1%) after COVID-19 pandemic. Analysis of pulmonary GGO patients showed similar results. The reason may be that younger patients, fewer comorbidities, less scope for removal of lung tissue, less trauma result in less postoperative complications [14].

The limitations of this study is that it is a single-center retrospective study with limited sample size, so larger sample size and multi-center study are needed to better reflect the current trend. Another limitation of this manuscript is the lack of long-term prognosis data for the patients and the absence of relevant outcome presentations. This article also has a limitation in that the changes in these data may be temporary and could have occurred solely due to the occurrence of COVID-19. Whether they have long-term significance would require further validation with more data.

In conclusion, we analyzed the changes in the composition ratio of patients underwent pulmonary surgery in Wuhan Tongji Hospital 1 year before and 1 year after the outbreak of COVID-19, and found that the number of young, non-smoking, non-tumor history, and female patients with lung adenocarcinoma increased after the outbreak of COVID-19, thus making recommendations for lung cancer screening. Through the results of this article, many potential low-risk female lung cancer patients have been identified. Additionally, there is an increase in young lung cancer patients under 50 years of age. These suggests that the current screening standards for lung cancer are not perfect, and some improvements need to be made, otherwise a large number of early lung cancer will not be timely diagnosed and treated, and the best time for surgery will be missed, resulting in poor prognosis. The paper also analyzed the changes in pathological types, stages, surgical ways and postoperative recovery of patients undergoing pulmonary surgery, which is conducive to better assessment of the challenges we are currently facing.

#### Statement

All procedures comply with the guidelines and regulations of the relevant Helsinki Declaration.

### Supplementary Information

**Additional file 1.**

## Data Availability

The article’s data will be shared on reasonable request to the corresponding author.
